# Comparison of analyses of the QTLMAS XIV common dataset. I: genomic selection

**DOI:** 10.1186/1753-6561-5-S3-S1

**Published:** 2011-05-27

**Authors:** Marcin Pszczola, Tomasz Strabel, Anna Wolc, Sebastian Mucha, Maciej Szydlowski

**Affiliations:** 1 Department of Genetics and Animal Breeding, Poznan University of Life Sciences, Wolynska 33, 60-637 Poznan, Poland; 2 Animal Breeding and Genomics Centre, Wageningen UR Livestock Research, 8200 AB Lelystad, The Netherlands; 3 Animal Breeding and Genomics Centre, Wageningen Institute of Animal Sciences Wageningen University, 6700 AH Wageningen, The Netherlands; 4 Department of Animal Science and Center for Integrated Animal Genomics, Iowa State University, Ames, IA 50011-3150, USA

## Abstract

**Background:**

For the XIV QTLMAS workshop, a dataset for traits with complex genetic architecture has been simulated and released for analyses by participants. One of the tasks was to estimate direct genomic values for individuals without phenotypes. The aim of this paper was to compare results of different approaches used by the participants to calculate direct genomic values for quantitative trait (QT) and binary trait (BT).

**Results:**

Participants applied 26 approaches for QT and 15 approaches for BT. Accuracy for QT was between 0.26 and 0.89 for males and between 0.31 and 0.89 for females, and for BT ranged from 0.27 to 0.85. For QT, percentage of lost response to selection varied from 8% to 83%, whereas for BT the loss was between 15% and 71%.

**Conclusions:**

Bayesian model averaging methods predicted breeding values slightly better than GBLUP in a simulated data set. The methods utilizing genomic information performed better than traditional pedigree based BLUP analyses. Bivariate analyses was slightly advantageous over single trait for the same method. None of the methods estimated the non-additivity of QTL affecting the QT, which may be one of the constrains in accuracy observed in real data.

## Background

An idea of genomic selection (GS) has been presented nearly a decade ago [1] and since that time it has been applied to plant [2] and animal breeding [3]. Together with an increased availability of dense marker assays, implementation of GS in breeding programs has become more popular [4,5] stimulating development of methods to estimate genomic breeding values.

Genetic basis of a phenotypic trait – its genetic architecture - is often complex. A particular trait may be, for example, controlled by many genes with small effects or by several major genes. Genes that control one trait may also control other trait(s), i.e. they are pleiotropic and the traits are genetically correlated. A gene variant may have an effect when it is inherited from a parent of one sex but not from the other (i.e. imprinting) or its effect will be present only when several alleles are in a particular combination (i.e. epistasis or haplotype effect). Genomic selection opens new opportunities in the analyses of complex traits.

A number of approaches have been developed to obtain direct genomic values (DGV) or genomic-enhanced breeding values (GEBV) [1,6]. Because number of markers is usually greater than number of genotyped individuals, predictions of individual genes are based on Bayesian model averaging, penalized regression, dimension reduction methods and algorithmic machine learning methods. .Several Bayesian models have been developed to model effects of individual loci. They differ in number of characteristics, including a prior distribution of number of QTL, their effects and assumption of homogeneity or heterogeneity of QTL variance (Table 1). Ridge regression (RR) and spatial regression are two types of panelized estimation, which assume homogenous variance across all markers. Double hierarchical generalized linear models (DHGLM) estimates marker-specific variances and can be solved by the iteratively weighted least squares. Partial Least Square Regression (PLSR) is an extension of the principal component analyses (PCA): the most systematic variations in marker data are decomposed into a small number of latent variables (principal components). This method reduces the dimensionality of the problem utilizing existing correlations between SNP [7]. GBLUP is an alternative, which treats the markers as a source of information on relatedness among individuals and models the sum of all QTL instead of individual loci. Some variants of GBLUP use preselected SNP to build relationship matrix for particular trait [1]. Some authors apply machine learning approaches (boosting, support vector), with hope that these methods better account for interaction between QTL [8].

**Table 1:** Bayesian models developed for genomic selection.

Feature Model	BayesA	BayesB	BayesC (=SSVS stochastic search variable selection)	BayesCpi
Probability for a locus to be a QTL	1	1-p	1-p	1-p
QTL-specific effect variance (variance heterogeneity)	Yes	Yes	No	No
Modelling of no-QTL	Not aplicable	Null variance	Tiny variance	Null variance
Estimated parameter				p(uniform prior)
Hyperparameters (assumed known)	df^1^, S^2^	df, S, p	df, S, p	df, S
Use Metropolis-Hastings sampler?	No	Yes	No	No

^1^df=degrees of freedom; ^2^S=scale parameter, the two parameters of scaled inverted Chi-square distribution (df, S) used as a priori distribution for QTL effect variance

For the XIV QTLMAS workshop, a dataset for traits with complex genetic architecture has been simulated and released for analyses by participants [9]. One of the tasks was to estimate DGV for individuals without phenotypes. The aim of this paper was to evaluate and compare results of different approaches used by the participants to calculate DGV.

## Methods

### Simulated data

Simulated, four-generation pedigree consisted of 3,226 individuals, descended from 20 founders, each mating resulted in 30 offspring. The last generation consisted of 900 young individuals with no progeny and no phenotypes. All 3,226 individuals had 100 Mb long genomes consisting of 5 chromosomes. In total, 37 out of 10,1031 single nucleotide polymorphism (SNP) markers were assumed to be QTL of which two had major effects. One of the simulated traits was a quantitative trait (QT) and the other one was a binary trait (BT). Heritability for QT, due to imprinting, was higher for males (0.52) than for females (0.39). True breeding values (TBV) for QT were calculated as a summation of effects of 30 additive QTL, haplotype effects (QTL pairs 31-32 and 33-34) and effects of imprinted QTL (for males only). Heritability for BT was 0.48. TBV for BT were calculated as a summation of effects of 22 additive QTL. Simulated pedigree, genome, marker and phenotypic data were made available for analyses. More detailed description of simulation can be found in [9] and the simulated dataset is available at http://jay.up.poznan.pl/qtlmas2010/dataset.html.

### Methods used by participants to estimate genomic breeding values

Eleven groups submitted their estimates of DGV. Participants applied several methods and often different variants of same method [8,10-17]. In total, they applied 26 approaches for QT and 15 approaches for BT (Table 2 and 3). The QT was analyzed by 11 groups, whereas BT by 6 groups. Ten groups used univariate models and two groups applied bivariate models. Bayesian models were used by five groups, machine learning was applied by a single group, and eight groups tested other methods.

**Table 2:** The comparison of the applied approaches used by participants for estimation of genomic breeding value of quantitative trait.

Approach no.	Authors	Method	Acc.		Reg. Coef.		MSD	Shared (%)	Loss (%)
			♂	♀	♂	♀			
1	Calus et al.[10]*	BayesA bivariate	0.85	0.84	1.06	0.91	45.4	17	14
2	Calus et al. [10]	BayeaA univariate	0.84	0.83	1.05	0.90	46.9	58	18
3	Calus et al. [10]	BayesC bivariate	0.87	0.89	1.01	0.88	42.4	71	10
4	Calus et al. [10]	BayesC univariate	0.86	0.87	1.01	0.89	44.1	68	12
5	Calus et al. [10]	GBLUP bivariate	0.83	0.81	1.07	0.90	47.8	57	19
6	Calus et al. [10]	GBLUP univariate	0.83	0.80	1.10	0.90	48.9	54	22
7	Calus et al. [10]	Pedigree-BLUP univariate	0.49	0.46	0.88	0.71	66.4	17	79
8	Calus et al. [10]	Pedigree-BLUP bivariate	0.50	0.47	0.88	0.72	66.8	23	62
9	Cleveland et al. [11]	BayesA_all ^1^	0.85	0.86	1.13	0.96	45.0	70	12
10	Cleveland et al. [11]	BayesA_s1^2^	0.49	0.52	0.94	0.91	63.4	26	63
11	Cleveland et al. [11]	BayesA_s2^2^	0.67	0.66	0.94	0.84	56.5	54	33
12	Coster and Calus[12]	PLSR^3^	0.76	0.73	9.05	7.31	76.4	16	83
13	Nadaf et al. [13]	BayesB	0.89	0.89	1.04	0.91	41.7	77	8
14	Nadaf et al. [13]	BayesB + Pedigree information	0.88	0.88	1.02	0.90	42.2	71	9
15	Nadaf et al. [13]	GBLUP + Pedigree information	0.81	0.80	1.09	0.92	49.2	56	21
16	Nadaf et al. [13]	GBLUP	0.82	0.80	1.12	0.92	49.1	71	23
17	Ogutu et al. [8]	Boosting	0.47	0.38	0.19	0.15	280.7	29	65
18	Ogutu et al. [8]	Support vector	0.69	0.63	1.54	1.20	48.3	49	36
19	Schulz-Streeck et al. [14]	Ridge regression	0.85	0.84	1.02	0.86	59.6	59	19
20	Schulz-Streeck et al. [14]	Spatial regression	0.83	0.81	1.08	0.88	46.4	63	19
21	Shen et al. [15]	DHGLM^4^	0.82	0.80	1.03	0.84	49.9	58	15
22	Sun et al. [16]	BayesCpi	0.89	0.89	1.05	0.91	41.6	77	8
23	Zhang et al. [17]	BayesB	0.89	0.89	1.05	0.91	42.0	74	8
24	Zhang et al. [17]	TA–BLUP–sub^5^	0.89	0.89	1.03	0.90	42.2	73	9
25	Zhang et al. [17]	TA–BLUP–all^6^	0.89	0.89	1.06	0.92	41.9	72	9
26	Zukowski et al.	GBLUP	0.58	0.59	1.12	0.96	87.0	41	38

^*^ Reference to applied method;^1^ with use of all markers in analyses; ^2^ with use of subset of markers in analyses; ^3^ Partial least squares regression; ^4^ Double hierarchical generalized linear models; ^5^ BLUP with trait specific matrix obtained with use of subset of markers; ^6^ BLUP with trait specific matrix obtained with use of all markers. Acc=accuracies of DGV (Acc.); linear regression coefficients of TBV on DGV; mean square differences (MSD) between TBV and DGV; percentage of IDs shared between the groups of young individuals selected on TBV and EBV (Shared) and percentage of loss of response to selection when 10% are selected based on EBV instead of TBV for quantitative trait (QT)

**Table 3:** The comparison of the applied approaches used by participants for estimation of genomic breeding value of binary trait.

Approach no.	Authors	Method	Acc.	Reg. Coef.	MSD	Shared (%)	Loss (%)
1	Calus et al. [10]*	BayesA bivariate	0.82	0.91	0.33	60	20
2	Calus et al. [10]	BayeaA univariate	0.73	0.89	0.47	53	28
3	Calus et al. [10]	BayesC bivariate	0.85	0.95	0.26	64	15
4	Calus et al. [10]	BayesC univariate	0.79	0.91	0.37	56	22
5	Calus et al. [10]	GBLUP bivariate	0.79	0.88	0.38	60	20
6	Calus et al. [10]	GBLUP univariate	0.72	0.83	0.49	52	29
7	Calus et al. [10]	Pedigree-BLUP univariate	0.52	0.71	0.74	30	52
8	Calus et al. [10]	Pedigree-BLUP bivariate	0.47	0.75	0.79	28	52
12	Coster and Calus[12]	PLSR^1^	0.72	0.78	1.40	20	71
13	Nadaf et al. [13]	BayesB	0.82	0.94	0.31	59	20
14	Nadaf et al. [13]	BayesB + Pedigree information	0.82	0.94	0.31	59	21
15	Nadaf et al. [13]	GBLUP + Pedigree information	0.71	0.84	0.50	51	30
16	Nadaf et al. [13]	GBLUP	0.71	0.84	0.50	51	29
21	Shen et al. [15]	DHGLM^2^	0.72	0.83	0.49	50	29
26	Zukowski et al.	GBLUP	0.56	0.81	0.69	38	47

^*^ Reference to applied method; ^1^ Partial least squares regression; ^2^ Double hierarchical generalized linear models.

### Comparison criteria

Five criteria were used to compare the applied genomic selection methods: (1) Accuracy being the Pearson correlation between true breeding values (TBV) and DGV. (2) Bias of estimates calculated as the linear regression coefficient (TBV = M + b*DGV +E) (unbiased estimates are expected to have regression coefficient of 1), (3) mean square difference (MSD) between TBV and DGV, (4) % of shared ID when selecting top 10% (45 males and 45 females) based on DGV vs. TBV, and (5) selection loss from selecting on DGV instead of TBV as a proportion of response using TBV. Due to a presence of imprinting, the average genetic values for males and females were different, and therefore, accuracies and regression coefficients for these groups were calculated separately.

## Results

### Accuracy

For QT, the accuracy was between 0.26 and 0.89 for males and between 0.31 and 0.89 for females (Table 2). Most of the approaches using Bayesian model averaging methods performed slightly better (average accuracy 0.68) than other methods. GBLUP yielded an average accuracy of 0.61 (after exclusion of the least accurate case of GBLUP). Traditional pedigree BLUP ignoring genomic data was only about half as accurate as the best approaches. No substantial differences between bivariate and univariate analyses were found. For BT, accuracy level was, generally, higher than for QT and ranged from 0.27 to 0.85 (Table 3). Similarly to the QT, Bayesian approaches were somewhat superior to other methods. The highest accuracy was reached by bivariate BayesC approach. Unlike QT, for BT bivariate analyses were considerably more accurate than univariate ones.

### Regression coefficient

For QT, regression coefficients ranged from 0.19 to 9.05 for males and from 0.15 to 7.31 for females (Table 2). Similarly to the previous criterion, the best performing approaches were Bayesian methods. For BT, regression coefficients ranged from 0.61 for some of GBLUP applications to 0.77 for bivariate BayesC (Table 3). Again for this trait bivariate analyses appeared to be better than univariate, which was not the case for QT.

### Mean square difference (MSD)

For QT, MSD for most of the approaches ranged from 42 to 63 (Table 2). Higher MSD were observed for one of the machine learning techniques - boosting – (280.7), one case of GBLUP analyses (87.0) and PLSR (76.4). These approaches were inferior in comparison to pedigree BLUP that yielded MSD of 66.4-66.8. For BT, MSD ranged from 0.26 for BayesC bivariate to 1.20 for PLSR. These results indicate that BayesC bivariate was superior to remaining methods (Table 3).

### Shared

For QT, percentage of ID shared between the groups of young individuals selected on TBV and DGV varied substantially and ranged from 16% (PLSR) to 77% (Table 2). The best three methods were: BayesB (74%-77%), BayesCpi (77%) and TA-BLUP (72%-73%). For pedigree BLUP only 17% (univariate) or 23% (bivariate) ID were shared with true top individuals. For BT, similar range of variation of shared ID was observed (from 20% with PLSR to 64% with bivariate BayesC) (Table 3). The best three approaches were: bivariate BayesC (64%), bivariate GBLUP (60%), and bivariate BayesA (60%). For pedigree BLUP only 30% (univariate) or 28% (bivariate) ID were shared.

### Loss

High percentage of shared ID, generally, was associated with low level of loss in genetic gain. For QT, percentage of lost response to selection when 10% are selected based on DGV instead of TBV varied from low (8%) to very high (83%). Pedigree BLUP resulted in 62% to 79% of loss, whereas approaches using genomic information, in general, resulted in smaller loss. Most of analyses using BayesB as well as BayesCpi and TA-BLUP appeared to be superior to other methods and caused only 8% to 9% of loss. Percentage of loss for most of the GBLUP approaches was close to 20%. For BT, loss in response to selection was, usually, at higher level. The smallest observed loss was 15% for bivariate BayesB and the biggest was 71% for PLSR. Pedigree BLUP caused from 74% to 79% of loss. Bivariate analyses were superior to univariate for both traits.

## Discussion

When phenotypes for young individuals are not available, the approaches that use genomic information had superior performance compared to the methods that were based solely on pedigree information. Use of genomic information, therefore, led to improved breeding value estimation, which was also found by others [1,18-24]. The traits simulated for the XIV QTL-MAS workshop differed with respect to complexity and a number of QTL. Effects of simulated QTL were unequal, some QTL had large effects and most of the other QTL had small effects on the simulated traits. All Bayesian model averaging methods had similar accuracy. These models were, furthermore, expected to achieve higher accuracy than GBLUP because of relatively small number of QTL [25]. GBLUP, however, was expected to capitalize on genetic relationships between training and validation sets [26]. We have found that these two groups of methods yielded similar accuracies. Bastiaansen et al. [23] who analyzed results of the previous QTLMAS workshop also reached a similar conclusion. Lack of apparent advantage in terms of accuracy of a single method across a range of traits was also shown in other simulation studies [27] and in real data, e.g. [3] and [28].

When the same approach is used by different researchers, similar results are expected, which was not always the case in our comparison. One GBLUP implementation, for example, was about 0.20 less accurate than other GBLUP analyses. This suggests that the methods may be very sensitive to data preparation and that their implementations may vary in performance.

Bivariate analyses, in general, performed better than univariate analyses for the same approach. This was expected as the two simulated traits were indeed genetically correlated. Differences between univariate and bivariate analyses were especially apparent for BT, for which phenotypes carry less information. More complex approaches, requiring initial estimation of marker effects or use of machine learning techniques applied to QT provided similar or inferior results to simpler methods.

## Conclusions

Bayesian model averaging methods predicted breeding values slightly better than GBLUP in a simulated data set, where traits had complex genetic architecture (epitasis, pleiotropy, and imprinting) and were affected by relatively small number of QTL. The methods utilizing genomic information performed better than traditional pedigree based BLUP analyses. Bivariate analyses were slightly advantageous over single trait for the same method. None of the methods estimated the non-additivity of QTL affecting the QT, which may be one of the constrains in accuracy observed in real data.

## Competing interests

The authors declare no competing interests. MS and MP preformed comparative analyses.

## Authors’ contribution

MP and TS drafted the manuscript. MS and MP preformed comparative analyses. AW MS TS SM and MP critically revised the manuscript and contributed to discussion of the results.
